# The effects of parental’s cultural and economic capital and parental support on being an elite scientists

**DOI:** 10.1371/journal.pone.0287967

**Published:** 2023-07-18

**Authors:** S. Koza Ciftci, Engin Karadag, Hatice Ergin-Kocaturk

**Affiliations:** 1 Department of Mathematics Education, Akdeniz University, Antalya, Turkey; 2 Department of Educational Administration and Supervision, Akdeniz University, Antalya, Turkey; 3 Boğaziçi University, İstanbul, Turkey; Shanghai Ocean University, CHINA

## Abstract

Despite the rapid increase in the number of scientists all over the world in recent years, very few scientists can achieve to be part of elite scientist’s category. Although there are many studies focusing on elite scientists, these studies generally do not focus on their childhood and parental background. In this study, which attempts to fill this gap, we focus on the cultural and economic capital of the families of elite scientists in Turkey and their parental support in childhood to analyze the roles of these variables in their being elite scientists. First, we assess the impact of cultural capital (institutional, objectified, and embodied), economic capital, parental support, and perceived academic success in basic education on the probability of becoming an elite scientist. Second, we analyze the differences among elite scientists to shed light on the gender gap in academia. We collected the data from 1,966 scientists working at 87 universities in Turkey through an online survey. Some of our main findings are as follows: (a) cultural capital, parental support, and academic success in basic education all have a strong positive effect on becoming an elite scientist; (b) objectified cultural capital has the highest impact in that an increase in this capital increases the probability of becoming elite scientists by 19%; (c) economic capital has no significant effect on elite scientists. Elite scholars have certain common characteristics, but significantly they are different from their average peers in terms of cultural capital and parental support and (d) elite female scientists have higher of cultural capital, economic capital, parental support, and academic success than elite male scientists. This finding supports the existence of the academic inequality and suggests that female scientists need higher cultural capital, economic capital, parental support, and perceived academic success to become elite scientists than their male counterparts.

## Introduction

In recent years, Turkey has become one of the few countries with the largest higher education capacity in Europe in that it had 203 universities, 8 million undergraduate students and nearly 200 thousand teaching staff [1] through the investments it made to raise the number of universities. The Turkish higher education system has grown rapidly in the last 20 years. So much so that 131 of these 203 universities were established in the last 20 years. This quantitative growth has led to an increase in the number of scientists which has led to a decrease in the number of publications per academic in Turkey. Based on the problem that scientists that operates within the system have a qualification issue as the cause of underdevelopment, we focused on Turkey’s elite scientists and their predictors of being elite. Following several previous studies focusing on the same theme to varying degrees [2–8], our aim was to explore the differences and similarities of childhood and parental characteristics of “elite scientists” (Different terms are used for highly published, cited, and internationally recognized and award-winning researchers in higher education studies: Productivity academic, competence academic, effective academic, elite scientist, tenure-track faculty, etc. In our study, although the term “elite” is a complex term with sociological and political meaning, we preferred to use the term “elite scientists” because it is the most frequently preferred term in the literature. Elite scientists’ “elite positions” are simply the result of their academic productivity. The term “average” was preferred for those other than these scientists.) with large-scale quantitative materials, based on the finding in many studies that socioeconomic origins of the parental shape educational outcomes from early childhood to adulthood.

The major research questions of the study are as follows: (*i*) How do elite scientists differ from average scientists in their cultural capital (institutional, objectified, and embodied), economic capital, parental support, and academic success in basic education? and (*ii*) What are the differences between male and female elite scientists?

One of the important predictors of income inequality is education [9, 10]. Similarly, SES is one of the most important factors that determine the quality of the education you will receive and your academic success [11, 12]. This mutual relationship creates a dead-end (contradiction) especially for developing countries. Families spend significant amounts of time and money to help their children upgrade. In the context of Turkey, as in many countries, education is seen as almost the only way to have a higher income. This puts families and children in a tough race. But questions such as “can this brutal war of parents and students make a difference in the end? Does the state support individuals with low SES?” occupy the country’s agenda. PISA and TIMSS research carried out in recent years show that Turkey carries the flag of first place in inequality. For this reason, in our study, we focused on how inequalities among scientists [13] who are known to differ from society socially and culturally affect their academic performance. The focus on the cultural and economic capital of families of elite scientists and parental support during childhood is interesting for several reasons. First, these scientists are pioneers in expanding the boundaries of knowledge in their field. Their human capital can provide great social returns by increasing potential knowledge spillovers to their colleagues. Second, the research questions can inform us about the validity of the belief that working at the universities is the profession of the elite. Finally, one of the important issues is whether the fame and career of this elite group is a trait they inherited from their families or is it their own potential. It is also hoped that the findings of the study will contribute to the existing studies on elite scientists and encourage researchers and policy makers to think more about the issue.

The study is organized as follows: Next section provides background information about the topic, including *Pierre Bourdieu’s theory of capital* and *parental support* and the qualitative and quantitative findings on elite scientists. The next chapter presents the methodology of the study including study design, participants, data collection tools and data analysis. The section the findings is divided into two subsections. The first one presents the findings obtained from the logistic regression (the effects of cultural, economic capital, parental support, and academic success in basic education on the probability of becoming an elite scientist) and the second subsection reports the results obtained from the analysis of gender differences among elite scientists. It was followed by the discussion and conclusion sections.

## Background

There are quite a variety of capital theories in the literature on intangible capital types, which include elements such as people’s working power, knowledge, ability and thinking ability. The most interesting of these theories belongs to Pierre Bourdieu. In Bourdieu’s theory of capital, he argues that there are various types of capital that individuals and groups own and use to gain advantage and power in society. In his seminal work, [14] distinguished three basic forms of capital that provide a better understanding of social change and the social distribution of power. According to his conceptualization, economic capital refers to economic resources (e.g., income, property). Social capital refers to resources available through social ties and membership in social networks. Cultural capital includes “instruments for the appropriation of symbolic wealth worthy of being sought and possessed” (p. 175). Bourdieu [14] stated that the relationship between the ownership and use of different types of capital is interdependent and reinforces each other. For example, individuals with economic capital are more likely to have access to high-quality education and cultural resources, which can help them accumulate more economic and cultural capital. In his later work, Bourdieu distinguished between three more specific forms of cultural capital: Embodied, institutionalized and objectified cultural capital [15].

### Cultural capital

Pierre Bourdieu introduced the concept of cultural capital which has been used in many studies as a starting point [16]. Cultural reproduction theory was originally developed as a tool to explain how children’s success at schools depends on the educational level of their parents [17]. Bourdieu, who first introduced the concept of capital language, replaced this concept with cultural capital in 1979 [18]. This argument about the advantages derived from cultural knowledge, habits, and tastes was expanded from the education system to the whole society, with an analysis of the lifestyles, tastes, cultural competences and participation in different groups, as well as their attitudes on cultural, moral and political issues in *Distinction* [15].

According to the theory, children of educated parents enjoy advantages not only because of the direct assistance they receive from their parents, but also because of their close familiarity with high-level culture such as the fine arts and classical music [19]. Again, [14] defined cultural capital as the familiarity with the dominant written cultural codes in a society and argued that cultural capital is an equivalent of the economic resources (economic capital) and social networks (social capital). Bourdieu argues that cultural capital can be transformed into economic and social capital as well as being a resource on its own [20].

In summary, in the most general sense, cultural capital refers to the cultural characteristics rewarded in fields such as education. However, it is possible to argue that this concept is used in different meanings both in Bourdieu’s studies and in other studies [16]. Studies that confined this concept to a narrow field such as fine arts have been severely criticized by researchers [21].

According to Bourdieu [14, 22], cultural capital exists in three forms: embodied (language, attitudes, preferences, etc.), objectified (cultural goods, books, works of art, etc.) and institutionalized (educational credentials). It may promote social reproduction in all three states. Cultural capital includes knowledge and social contexts, parental’s educational background, relatives and social environment relations, and social status. How students socialize can be considered as an embodied form of cultural capital. This situation occurs through students’ social environments and educational qualifications. Parents transfer their cultural capital to children either by unknowingly exposing them to objectified and embodied cultural capital at home or by actively investing their cultural capital in transferring their cultural capital to children. Over time, children internalize the cultural capital of the parents, which becomes an integral part of their parenting and behavior, namely what Bourdieu calls habitus [20]. Habitus is embodied intellectual tendencies that together with social position form a social-relationship structure. Habitus is understood as a system of internalized schemas that allow us to understand the construction of all thoughts, perceptions and actions belonging to a particular culture [23].

In this study, cultural capital is as the institutionalized cultural capital dimension that measures the educational and professional levels of the scientists’ parents, the objectified cultural capital dimension that measures the number of books and art works in the home where the scientists grow, and the materialized cultural capital dimension that measures the participation of scientists in social and cultural activities in their childhood.

### Economic capital

In the context of Pierre Bourdieu’s theory of capital, economic capital refers to the resources that an individual or group has access to through their economic position such as income, wealth, property, and other tangible assets. Bourdieu argued that economic capital is only one of the various forms of capital owned by individuals, and that these different forms of capital interact with each other to shape the individual’s social position and social mobility opportunities. According to Bourdieu, economic capital is often the most visible and most valuable form of capital in capitalist societies, but it is not the sole or most important determinant of social status and power.

Many studies have revealed that parental economic capital affects their children’s educational attainment in various ways. The larger the economic capital, the more families invest in school education [23]. Thus, families with strong economic capital aim to reproduce cultural capital by sending their children to schools of their choice. Because as the privileges of the social class increase, the cultural capital also increases at the same rate. In summary, parents with high economic capital, usually university graduates and high-status occupations, (*i*) develop advantageous cultural and social capital for their children, (*ii*) prioritize organized activities that facilitate educational success, and (*iii*) spend more time for engaging in developmental activities that take advantage of their children’s success [24–26]. We therefore consider how parental economic capital relates to their children’s likelihood of becoming elite scientists. In our study, the economic capital was functionalized as a single dimension based on objective measurements such as the economic status of the scientists’ families in their childhood, the number of siblings and the place of residence in their early childhood.

### Parental support

Parental support in education refers to the participation of parents in their children’s educational journey. This includes providing support and encouragement for their children’s academic progress, attending parent-teacher conferences, volunteering at school events, and helping with homework or study. Parental support may also include advocating for their children’s educational needs and communicating with teachers and school staff to ensure their children receive the necessary resources and support to succeed [27–29]. Policy makers, school leaders, and teachers in many education systems often advocate that parents should work with schools and be involved in their children’s learning [30]. Not surprisingly, expectations for parent involvement in their children’s education have motivated a significant body of research examining the relationship between parent involvement and student achievement [28, 31, 32]. Some studies found that lower SES parents were not as involved as parents from more advantaged social backgrounds [33, 34]. Again, many studies have determined the effects of parental support on academic success. For these reasons, schools today take various steps to increase parental support. However, the way of this support- parental’s expectations of the child, the child’s potential, helping with homework, being interested in school duties, and frequency of school visits- is quite important [28].

In our study, we consider how being an elite scientist is systematically related to parental support, and how the role of parental support varies across broad disciplines. However, in our study, since we could not measure the parental support of the scientists objectively, we considered it as a whole and it was functionalized as a dimension by questioning the participants’ families’ support. This situation creates a limit for the study.

## Research questions

In organizations with a high level of human capital such as higher education institutions, human capital regulates the interaction between employees and provides a strong commitment to organizational values and goals. In the absence of human capital, organizational assets cannot have any effectiveness which causes difficulties in cultural and economic development processes. The existing evidence (1) supports the impact of human capital in improving employee job performance. However, little is known so far about the cultural capital that both ‘elite’ and ‘average’ scientists have acquired from their families. In this context, we focused on the concept of elite scientists, which is thought to be not sufficiently mentioned in the related studies. This focus leads to the study’s first research question, **RQ1**, as follows: How do elite scientists differ from average scientists in their cultural capital (institutional, objectified, and embodied), economic capital, parental support, and scientist success in basic education? To validate this research question, we checked for the influence of factors assumed to affect their research productivity/output. The second research question, **RQ2**, is as follows: What are the differences between male and female elite scientists in terms of them of cultural capital (institutional, objectified, and embodied), economic capital, parental support, and scientist success in basic education?

## Method

As explained earlier, our analysis will focus on how both the cultural and economic capital of families and personal factors influence becoming an elite scientist. We begin this section with details on our elite scientist admission requirements. We then describe our measures and analysis of the cultural and economic capital and personal factors of the scientists in our sample.

### Elite scientist

This study was conducted on elite and average scientists. In the study, we defined elite scientists as those who are successful in their field, won awards and have achieved international and measurable success in their fields. We selected elite scientists using the award data from the Turkish Academy of Sciences, Science Academy and TUBITAK [35]:

- Turkish Academy of Sciences Outstanding Young Scientists Awards (GEBİP): The GEBİP award program, launched in 2001, is one of the first of its kind in the world. The objective of TÜBA-GEBİP is to foster young, outstanding scientists who are at the stage of establishing their own research programs in Turkey after finishing their post-doctoral research activities. TÜBA supports these scientists for a period of three years and helps them set up their own research groups at a stage when they need incentives. The GEBİP program aims to set up a kind of Young Academy of scientists under the age of 40. Aside from a grant provided for a period of three years, a member of the Academy is assigned as the mentor, and an environment of solidarity and interaction is created through joint meetings held with Academy members. The GEBİP Programmed is conducted by the GEBİP Main Committee and the GEBİP Field Committees set up by the Presidency of the Academy. Applications may be in person, by the President of the related university or the highest administrator of the related institution, or by a Principal or Honorary member of the Academy. The selection procedure among GEBİP candidates has very high standards. First, applicants prepare detailed dossiers including their international publications in refereed journals, books edited, ongoing research projects, and proposed research program, plus three letters of reference. Second, juries in four different scientific fields (field committees) evaluate and grade the applicants according to their dossiers and three referee reports. In the final stage, applicants who become GEBİP candidates according to their grade points are invited to an interview. Every six months, the GEBİP awardees are expected to submit a detailed report to their mentor about the progress of their scientific research and publications. The mentors convey their evaluations about the GEBİP awardees’ scientific activities to the Academy Council at the end of every six months. Based on these evaluations, the Academy Council decides to continue, stop, or end the payment of the award. The limit of the award period is three years.
- Science Academy’s Young Scientist Awards Program (BAGEP): A top priority for the Science Academy is encouraging the youth to engage in good science and rewarding the best examples. To choose and reward the best young scientists and to support them in conducting new studies, an award program was initiated in the year 2013. Science Academy runs this program not with government funding, but with the support of the society at large. The award-winning young scientists are granted 20,000 TL per year for a period of two years to support them in their research. The objective here is to reward the most brilliant and promising young scientists with a prestigious grant which will help them further their studies. Scientists younger than 40 are eligible for the award. Candidates may apply in person for the award. Science Academy members, faculty members, deans and presidents of universities may also nominate young scientists.
- TUBITAK (The Scientific and Technological Research Council of Turkey) Encouragement Award: It is given to scientists residing in Turkey, who have proven that they have the qualifications to make significant contributions to science at an international level in the future, with their work, and who are under the age of 40 on the first day of the year in which the award is given.

The main reasons for determining these three institutions are; (i) they are members of many internationally respected scientific organizations (All European Academies, The InterAcademy Partnership, Association of Academies and Societies of Sciences in Asia, International Science Council, International Union of Academies The InterAcademy Council, International Human Rights Network of Academies and Scholarly Societies); (ii) they are not public institutions (Science Academy); (iii) they ensure diversity of awarding institutions; (iii) awarding programs have been in place for at least 10 years due to the exclusionary age factor in awards; (vi) they do not serve specifically to a particular science discipline and (vii) allow researchers to apply individually for the awards. The number of scientists who have received any of these awards in Turkey is quite low (May 2022: 693 number). Therefore, these scholars can be termed elite. Other scientists are those who work at the universities who have similar gender, age, scientist title, and research fields, and are called “average” scientists by Prpić [8].

### Participants

In the first stage of our study, we obtained the list of elite scientists from the official web pages of “Turkish Academy of Sciences”, “Science Academy” and “TUBITAK”. When we combine the award recipients from more than one organization, the number of elite scientists is 693. Of the elite scientists, 220 (32%) were female, 473 were male (68%); 87 were from health sciences (13%), 163 were from social sciences (24%), 211 were from engineering (30%) and 232 were from science and math (33%).

In the second stage, we used a database provided by the Council of Higher Education (CHE Academic), which presents all scientists, including elite scientists in Turkey. The database contains information such as scientists’ names and surnames, contact information, details of education degrees, scientific disciplines, publications, awards, etc. We updated the missing information in the database from the official web pages of the universities where the scientists work. In our study, we conducted a large survey of scientists in Turkey in two parallel groups. In the first group, 693 “elite” scientists from 87 universities, 59 of which are state and 28 of which are foundation received that questionnaire. In the second group, we sent the online questionnaire in the summer of 2022 to 8,754 “average” scientists with similar qualifications (science discipline, age range, university, city, etc.) with elite scientists. The number of returned questionnaires was 160 (23% response rate) in the elite scientist group and, 806 (21% response rate) in the average scientist group; and the total is 1,966. Overall, response rates were similar within the two groups. In terms of gender, 578 (29.4%) of the participants were female and 1,388 (70.6%) were male. The participants were between 29 and 52 years old ([Elite scientists *M* = 41.20; *SD* = 3.34], [Average scientists *M* = 42.92; *SD* = 4.63], [Total *M* = 42.01; *SD* = 4.17). The majority of the participants are professors (44%) and associate professors (41%). One third of the scientists participating in the study are from the field of science and math (33%). Similar groups were formed in our study following with the argument that cultural capital contributes significantly more strongly to the performance in “soft” disciplines [14] and in order to ensure the representativeness of the sample. As it can be seen in Table 1, both elite and average scientists are very similar groups to the population in terms of gender, age, scientist title and research areas among in the group and inter groups. This indicates that there is no potential selection bias.

**Table 1:** Information of the scientists’.

Variables	Elite Scientists		Average Scientists		Total	
	n	%	n	%	n	%
**Gender**						
FEMALE	46	28.7	532	29.5	578	29.4
MALE	114	71.3	1274	70.5	1388	70.6
**Academic Title**						
PROFESSOR	72	45.0	806	44.6	878	44.7
ASSOC. PROFESSOR	64	40.0	756	41.9	820	41.7
ASSISTANT PROFESSOR	24	15.0	244	13.5	268	13.6
**Science Area**						
SOCIAL SCIENCES	37	23.1	418	23.1	455	23.1
SCIENCE AND MATH	54	33.8	599	33.2	653	33.2
ENGINEERING	49	30.6	547	30.3	596	30.3
HEALTH SCIENCE	20	12.5	242	13.4	262	13.3

### Measurement

At the beginning of the study, the “*Social and Human Sciences Scientific Research and Publication Ethics Committee*” approved the study protocol. We developed a survey questionnaire containing demographic questions and then we contacted scientists. We first explained the purpose of the study to the scientists, informed the participants about the confidentiality of the data, the volunteering and anonymity of the participation, and informed consent forms. The link of the survey questionnaire was sent to the scientists who were volunteer to participate the study, and they were asked to answer the online survey questionnaire. Written informed consent was obtained from all participants for participation in the study. It took the participants approximately 10 minutes to complete the survey questionnaire.

#### Measurement of the cultural capital pattern

In the survey questionnaire cultural capital pattern is made up of three dimensions and ten items with different formats. The first four items measure the **Institutionalized Cultural Capital**. The items about this dimension are formed in a scale structure. The institutionalized corporate capital scores of the participants were calculated using “Equality 1”. ISCO-08 (2008) rating was employed for the participants’ parental professional skill scores. For the parental education levels, the level rating of 0–8 of the International Standard Classification of Education (ISCED) of the UNESCO 2011 version was used. These variables were not used in the measurement of the Institutionalized Cultural Capital dimension, since all the participants in the study had the same education level and occupation.


((mother’sprofessionalskilllevel+mother’seducationalbackground)/2)+((father’sprofessionalskilllevel+father’seducationalbackground)/2) Eq 1

Two items in the survey questionnaire are about the **Objectified Cultural Capital**. The related items are as follows: I1: “How many books (excluding textbooks) were in your home in your childhood?”, I2: “How many works of art (paintings, sculptures, etc.) were in your home in your childhood?” The options given for the first item are as follows: “10 books or less”, “11–25 books”, “26–100 books”, “101–200 books”, “201–500 books” and “500+ books”. The options given for the second item are as follows: “None”, “1–2 works”, “3–5 works”, “6–10 works” and “more than 10 works”. For the participants’ objectified cultural capital (childhood/history) scores, first the lowest book/artwork group was scored from 0 to 5. Then, objectified cultural capital scores were obtained by summing the scores of the two questions for each participant.

The four items of the questionnaire measure the **Embodied Cultural Capital** dimension of cultural capital. The items measuring this dimension are as follows: I1: “How often did your parental members read books in your childhood?”, I2: “What was your level of participation in extracurricular activities in your childhood?”, I3: “How often did you discuss political or social issues with your parents in your childhood?” and I4: “How often did you discuss books, movies, or television shows with your parents in your childhood?”. The items are answers using a seven-point Likert scale (1 = “Never or Almost Never” to 7 = “Pretty much”).

#### Measurement of the economic capital pattern

In the study, the economic capital pattern consists of one dimension and three different types of items were used to measure it. The items measuring the economic capital structure are as follows: I1: “How would you describe your parental economically in your childhood?”, I2: “How many siblings do you have?” and I3: “In what kind of settlement did you spend your early childhood (0–7 years)?” The first item is answered using the options of “low”, “below medium”, “medium”, “above medium”, “high” and “very high”. The second item has the following answer options: “I don’t have any”, “I have 1 sibling”, “I have 2 siblings”, “I have 3 siblings” and “I have more than 4 siblings”. The answer options for the third item are as follows: “village”, “town”, “district” and “province”. For the economic capital scores of the participants, starting from the lowest economic level 1 to 6 the number of siblings was scored from 1 to 5 from the highest to the lowest, and from 1 to 5, starting from the settlement “village” to the “provincial center”. Then, the economic capital scores were obtained by summing the scores of the three questions for each participant.

#### Measuring parental support

The parental support structure consists of one dimension and is measured through four items. These are given as follows: I1: “What is the level of educational support of your parents in basic education?”, I2: “What is the level of support for your scientist career?”, I3: “How much did your parents’ occupations/works affect your interest in science?” and I4: “To what extent were your parents supportive of you being a scientist?” These items were answered using a seven-point Likert scale (1 = “Never or Almost Never” to 7 = “Pretty much”).

#### Measuring academic success

In the study, the academic success was measured with a single item. It is given as follows: I1: “How would you describe yourself as a student academically in your childhood?” It is answered using a seven-point Likert scale (1 = “Very Unsuccessful” to 7 = “Very Successful”).

### Analysis

The methods used in this study are confirmatory factor analysis (CFA), correlation, MANOVA, and logistic regression analysis using explanatory factor analysis (EFA) and partial least squares (PLS). The “two-stage” approach was adopted because the embodied cultural capital, economic capital, and parental support structures are secondary structures. First order latent constructs were tested using EFA with varimax rotation. Second, it was evaluated using convergent validation and discriminant validation to check the validity of the measurement model. Cronbach α and composite reliability (CR) were used to test the reliability of the measures.

In the study, the relationships between the participants’ cultural capital (institutional, objectified, and embodied), economic capital, parental support and academic success scores were examined by correlation. Logistic regression analysis was used to test the theoretical model. In the theoretical model, it was assumed that cultural capital (institutional, objectified, and embodied), economic capital, parental support, and academic success had an impact on the award-winning status of scientists. At this stage, a statistically compatible model was obtained in which the relations between the variables were defined. In the data analyzes performed in this study, the significance was determined as *p* < .05. Finally, the statistical differences of the cultural capital, economic capital, parental support, and academic success scores of the award-winning academicians according to their gender were examined by multi-directional analysis of variance (MANOVA). Data were screened for the assumptions of parametric statistics. Normality, homogeneity of variances, and linearity assumptions for each cell were tested at multivariate level. In addition to, multivariate test statistics values, *F*s, and statistical significances, effects sizes (*η*^2^) and power estimates were reported. Effect sizes were reported as eta-squared [36]. As suggested Cohen [37], 0.01 is a small effect, 0.06 a moderate effect, and 0.14 is a large effect. In the data analyses carried out in the current study, significance was set at *p* < .05. The Statistical Package for Social Sciences (SPSS) 23.0 was used to code and analyze the data.

## Results

### Instrument validity and reliability

Exploratory factor analysis was used to confirm the one-dimensionality of the measurements before analysis. Institutionalized and objectified cultural capital and academic success constructs were not included in the EFA as they were formed from a single formative score as explained in the measurements section. It was understood that exploratory factor analysis could be performed with the results of the KMO, and Bartlet test analyzes of the firstly collected data. Afterwards, when EFA was performed with varimax fundamental axis rotation, it was determined that the factor loading of all items was above |.50| and only one factor was loaded (Table 2).

**Table 2:** Exploratory factor analysis results.

	FL	Com.
**Embodied Cultural Capital**		
** 1.**How often did your family members read books in your childhood?	.826	.800
** 2.**What was your level of participation in extracurricular activities in your childhood?	.814	.715
** 3.**How often did you discuss political or social issues with your parents in your childhood?	.763	.659
** 4.**How often did you discuss books, movies, or television shows with your parents in your childhood?	.665	.574
**Economic Capital**		
** 1.**How would you describe your family economically in your childhood?	.774	.609
** 2.**How many siblings do you have?	.737	.576
** 3.**In what kind of settlement did you spend your early childhood (0–7 years)?	.590	.514
**Family Support**		
** 1.**What is the level of educational support of your parents in basic education?	.877	.810
** 2.**What is the level of support for your academic career?	.838	.803
** 3.**How much did your parents’ occupations/works affect your interest in science?	.588	.540
** 4.**To what extent were your parents supportive of you being a scientist?	.554	.559

***Note*:** FL = factor load; Com. = communality

Based on the EFA, it can be said that the constructs produce AVEs between 0.50 and 0.69. Therefore, all constructs have convergent validity. The Cronbach α reliability coefficients of the measurement models ranged from .72 to .79; CR coefficients ranged from 0.51 to 0.90, with good to very good reliability. Therefore, questions in each construct are highly correlated, indicating that they measure the same latent construct (Table 3).

**Table 3:** Reliability, convergent validity and unidimensionality.

Dimensions	Items	AVE	α	CR	KMO	D.
**1-**Embodied Cultural Capital	4	.59	.79	.85	.72	1
**2-**Economic Capital	3	.50	.75	.64	.64	1
**3-**Family Support	4	.53	.77	.81	.72	1

***Note*:** AVE = average variance extracted; α = Cronbach’s α; CR = composite reliability; KMO = measurement of suitability of the Kaiser–Meyer–Olkin sample; D. = dimensionality

### Common method bias

Harman’s single factor test was used to control for common method bias [38]. All dependent and independent variables were subjected to explanatory factor analysis, and while the factors together accounted for 63.24% of the total variance, the first factor explained only 15.17%. These findings indicate that there is no problem in the dataset indicating common method bias [39].

### Descriptive findings

Elite and average scientists were described as independent variables of the study (see Table 4). The mean scores of cultural capital, economic capital, parental support, and academic success in childhood of elite scientists are significantly higher than those of other scientists. Before testing the theoretical models developed in the study, the correlation coefficient of the relationships between the scores of the independent variables was examined. The results suggest that there is a positive significant correlation between some variables.

**Table 4:** Descriptive statistics of the study variables.

Dimensions	*M*	*SD*	*p*	*Hedges’ g*	1	2	3	4	5	6
**1-**Institutionalized CC.	6.36 (4.77)	3.01 (2.98)	< .01	.52	-	(.60*)	(.20**)	(.58**)	(.48**)	(.03)
**2-**Objectified CC.	4.05 (2.77)	2.20 (2.33)	< .01	.55	.51**	-	(.25*)	(.49**)	(.48**)	(.07*)
**3-**Embodied CC.	20.52 (18.34)	3.60 (4.39)	< .01	.50	.19*	.10	-	(.24**)	(.20**)	(.16**)
**4-**Economic Capital	10.42 (9.33)	2.52 (2.80)	< .01	.39	.66**	.36**	.12	-	(.42**)	(.02)
**5-**Parental Support	24.62 (20.13)	5.93 (5.95)	< .01	.75	.44**	.24**	.26**	.29**	-	(.12**)
**6-**Academic Success	6.09 (5.83)	1.26 (1.24)	< .01	.20	.22**	.10	.02	.09	.07	-

***Note*:** CC. = Cultural Capital; *p < .05; **p < .01; Elite scientists (Average academics)

### Logistic regression model

We set up a logistic regression model to determine the effects of cultural capital (institutional, objectified, and embodied), economic capital, parental support, and perceived academic success on the probability of becoming an elite scientist. As a result of the analysis, the Omnibus test results, which showed the general suitability of the model, indicated that the general significance of the model or the goodness of fit was statistically significant (*X*^*2*^ = 129.72; *df* = 7; *p* < .001). In addition, it is also found that the logistic regression model estimated based on the results of the Hosmer and Lemeshow test was suitable for the data (*X*^*2*^ = 3.165; *df* = 8; *p* = .924).

The overall fit of the model was good, and the independent variables in the model explained 34.9% (Nagelkerke R^2^) of the variability of elite scientists. The sensitivity rate, showing how sensitive the established logistic regression model was in detecting true positives, is found to be 86.2%. This shows that in the real situation, about 86% of elite scientists are predicted in a correct manner. The specificity rate, which shows how sensitive the test is in detecting true negatives, is found to be 99.5%. In other words, the model correctly predicted about 99% of the average scientists in the real situation. The correct classification rate of the model was determined as 91.7%. This shows that the model correctly predicts the elite status of approximately 92% of the scientists sampled. As a result, it can be argued that the classification power of the model is quite good.

When the coefficient estimates and odds ratios of the logistic regression analysis are examined, there appear six factors that have a significant effect on receiving rewards (see Table 5). When all other variables are controlled, the increase in the institutional cultural capital of scientists increases the probability of being an elite scientist by 14% (OR = 1.14, 95% CI [1.05–1.23]). Similarly, an increase in scientists’ objectified cultural capital increased the probability of elitism by 19% (OR = 1.19, 95% CI [1.09–1.30]); 8% (OR = 1.08, 95% CI [1.04–1.11]); an increase in parental support increases the probability of elite by 10% (OR = 1.10, 95% CI [1.07–1.13]) and an increase in academic success increases the probability of elitism by 19% (OR = 1.19, 95% CI [1.02–1.39]). Therefore, it is safe to state that there is a positive relationship between cultural capital, parental support and academic success and elite scientists. However, the variable of elite scientists and economic capital is found to be not statistically significant (*p =* .361).

**Table 5:** Logistic regression model coefficients.

Variable	*β*	SE	Ward	*p*	Exp(β) (Odds)	95% CI
**1-**Institutionalized CC.	.13	.04	11.27	.001	1.14	1.05–1.23
**2-**Objectified CC.	.17	.04	15.17	.000	1.19	1.09–1.30
**3-**Embodied CC.	.07	.01	24.26	.000	1.08	1.04–1.11
**4-**Economic Capital	.04	.04	.83	.361	1.04	.95–1.14
**5-**Parental Support	.10	.01	34.58	.000	1.10	1.07–1.13
**6-**Academic Success	.17	.07	5.15	.023	1.19	1.02–1.39
**Constant**	-3.99	.67	34.74	.000	0.01	

CC. = Cultural Capital; Dependent variable: Elite scientists (Binary; Yes = 1 and No = 0)

The results of the study indicate that the degree of these possibilities differs among the fields of the scientists. For example, the increase in the institutional cultural capital of scientists increases the probability of being an elite scientist by 16% (OR = 1.16) in social sciences, by 22% in science-mathematics (OR = 1.22), by 9% in engineering (OR = 1.09), and by 23% in health sciences (OR). = 1.23). The increase in the objectified cultural capital makes them more likely to be elite scientists: It is found to be 13% in social sciences (OR = 1.13), 29% in science-mathematics (OR = 1.29), 18% in engineering (OR = 1.18), and 9% in health sciences (OR = 1.09). The increase in the embodied cultural capital makes them more likely to be elite scientists: It is found to be 7% in social sciences (OR = 1.07), 14% in science-mathematics (OR = 1.14), 9% in engineering (OR = 1.09), and 3% in health sciences (OR = 1.03). The increase in parental support increases the probability of scientists’ elitism by 8% (OR = 1.08) in social sciences, 15% (OR = 1.15) in science-mathematics, 8% (OR = 1.08) in engineering, and 13% (OR = 1.13) in health sciences. The increase in parental support increases the probability of scientists’ elitism by 25% (OR = 1.25) in social sciences, 1% (OR = 1.01) in science-mathematics, 68% (OR = 1.68) in engineering, and 6% (OR = 1.06) in health sciences. However, the variable of economic capital is found to be not statistically significant in any area (*p >* .05).

### Gender differences among elite scientists

Significant correlation coefficients between the variables are found and they suggest that these variables are related to each other (Table 4). It is recommended to perform a single multiple variance analysis instead of a separate t-test for the variables that are theoretically and statistically related to each other. For this reason, the statistical differences of six variables based on gender were analyzed using the multi-way analysis of variance (MANOVA) (see Table 6). The MANOVA analyses were performed on a linear combination of six variables. Non-orthogonal pattern adjustment was made due to differences between cells. The data show that the variance-covariance homogeneity assumption required for multivariate analysis of variance is met (Box *M* = 14.457, *F* = 0.14, *p*>.05). In addition, the general homogeneity test for regression and the regression homogeneity general test for MANOVA indicate that these assumptions are fulfilled (*p* > .05). As a result of the analysis, a significant multivariance effect was found for gender (*Wilks’ λ* = .88, *F* = 2.28, *p <* .01, partial *η*^2^ = .12). The relationship between gender and combined dependent variables is found to be moderate. As the overall MANOVA differed significantly for gender, the nature of this difference was examined in more detail. Elite female scientists score higher than elite male scientists on cultural capital (institutional, objectified, and embodied), economic capital, parental support, and academic success. While the effect size of gender on cultural capital (institutional, objectified, and embodied), economic capital, parental support and academic success is statistically significant, it is in fact medium and minimal (partial eta-square = .06, .03, .04, .09, .06 and .03, respectively). These findings show that women need higher cultural capital (institutional, objectified, and embodied), economic capital, parental support, and academic success to become elite scientists compared to men.

**Table 6:** Variable differences in gender context.

Dependent variable	III Type Sum of Squares	*df*	Mean Squares	F	*p*	Eta-square
**1-**Institutionalized CC.	919.30	1	919.36	109.81	.000	.06
**2-**Objectified CC.	297.00	1	297.00	56.15	.000	.03
**3-**Embodied CC.	2737.02	1	2737.02	65.46	.000	.04
**4-**Economic Capital	1202.17	1	1202.17	166.32	.000	.09
**5-**Parental Support	3675.69	1	3675.69	109.80	.000	.06
**6-**Academic Success	88.77	1	88.77	58.82	.000	.03

CC. = Cultural Capital

### Randomization tests

Analyses in the study were carried out using the data obtained from a non-random sample. The randomization tests were performed to support generalizability of the findings beyond the research sample. In this context, 5000 bootstrap replicates were used to test their effects on the working model. When the means, standard errors, 95% CIs, significance levels, and the directions of the relationships are examined, it is seen that the bootstrapped samples are close to each other.

## Discussion

Many individuals begin to think about being an academic from early childhood. At an early age, students with academic inclinations may set goals for becoming a professor at a prestigious university [40]. Previous studies have focused on the impact of post-doctoral developments on the success of scientists at universities where they hold doctorates. In this study, we focused on the childhood and families of “elite” scientists. Moreover, and more importantly, it focused on the cultural and economic capital of the scientists’ families and their parental support during childhood and evaluated the impact of these variables on their being elite scientists. The model developed is new in the methodology of the studies on the topic. In RQ1, we analyzed the problem of how “elite” scientists differ from “average” scientists in their cultural capital (institutional, objectified, and embodied), economic capital, parental support, and academic success in basic education. According to the logistic regression model, the increase in the institutional cultural capital of the scientists increases the probability of being an elite scientist by 14%. The increase in their objectified cultural capital increases this probability by 19%, while the increase in their embodied cultural capital increases this probability by 8%. This finding supports the cultural capital theory, which is based on the assumption that individuals’ home environment in early childhood, their parents’ education levels, attitudes and behaviors are the source of their success [41]. The effects of all three dimensions of cultural capital on elite scientists is as follows, ranging from large to small: objectified cultural capital, institutionalized cultural capital, and embodied cultural capital. These results showed that the cultural background of the scientists which they inherit from their families can significantly affect their academic performance. Academic performance is not just one area of technique and skill, but also requires a range of experience with acceptable social norms and postures in the specific settings of the professional field [41]. These findings are consistent with the previous one (for instance, [42]). For example, the previous findings reveal that exposure to and participation in actions that are characteristics of cultural capital also enable access and mobility within a narrow social space [43, 44]. It has also been shown that cultural capital has a predictive effect on the future of individuals’ cultural capital in science [45–49]. Again, the results are quite consistent with Bourdieu’s theory which asserts that cultural capital is not an academic but an intellectual competence. Scientists, who have had high cultural capital gains since their childhood, rise to the level of elite scientists when they process their academic skills with their intellectual knowledge. In this respect, one of the ways to increase academic performance may be to improve the cultural gains.

Another result regarding the cultural capital result obtained in the study is that parental support in childhood has a significant effect on the promotion of scientists to the elite category. These two results are compatible with the cumulative advantage theory [50] which argues that successful and supported children from early childhood education will be more successful in the future. It can be said that elite scientists have the capacity to work hard and pursue long-term goals partly due to the privilege they bring from their families [51].

One of the most interesting findings obtained in the study is that the families of the “elite” academicians and the “average” academicians have similar economic capitals. This result differs from some of the previous findings. Positive effects of economic capital on a children’s education have been reported in the former studies [52–55]. This difference is due to the socio-demographic and economic structure of Turkey. Parents of “elite” scientists are mostly professional. Among them is teaching profession. To answer the question of why scientists whose families’ teachers are breaking the “rule”, it is necessary to look at Turkey’s teacher training policies. Since the establishment of the Republic of Turkey, to train teachers, successful students with low SES and generally living in rural areas have been selected from primary school, and in the following years from secondary school, and provided boarding education. Although this policy has been abandoned in recent years, it can be said that this situation creates a higher class of teachers in terms of cultural capital compared to the general society.

However, teachers’ salary has never exceeded certain standards. Even if both parents were teachers, the parental always remained at the middle-lower income level. This has led to the formation of a class with low income but high cultural capital. Today it is the profession group that receives the lowest wage among professional occupational groups. For example, while the minimum wage is 8,506 TL in 2023, the teachers’ salary is nearly 11,408 TL. For this reason, it is an expected result that there is no difference in the economic capital of elite scientists, whose parents are teachers and average scientists.

When the backgrounds of the “elite” scientists are examined, it is seen that the majority of them are graduates of Anatolian High School and Science High School, which provide education in a foreign language after primary school, and very few of them are private school graduates. Until a decade ago, very small portion of K-12 education in Turkey took place in private schools. After primary or secondary school, many successful students were able to attend quality public schools of which medium of instruction is English. In addition, special conditions were sought for teachers working in these schools. Moreover, students who wished could continue their education as a boarding student. This provided an equal opportunity for the relatively small minority who were admitted to these schools at an early age. Therefore, it shows that parents who attach importance to education and have high cultural capital (low economic capital) have their children received their basic education in Turkey’s most distinguished educational institutions. Today, parallel to the privatization of education, privileges regarding foreign language education in these schools have been abolished, the number of schools has been increased and teacher selection policies have been abandoned. Again, today, the only chance of students to get a qualified and good foreign language education is private schools, and families that do not have high economic capital do not have the chance to send their children to these schools. This situation makes us think that economic capital will have an impact on being an elite scientist in Turkey in the future.

To shed light on the gender gap among the scientists, the study also focuses on the differences among elite scientists. In this context, we analyzed the problem of how different the of “elite” female scientists and “elite” male scientists in terms of cultural capital (institutional, objectified and embodied), economic capital, parental support and academic success in basic education differ which was covered by the RQ2. The results revealed that elite female scientists have higher of cultural capital, economic capital, parental support, and academic success than elite male scientists. This finding supports the existence of a significant academic inequality in science and contributes to the view that female scientists need higher cultural capital, economic capital, parental support, and perceived academic success to become elite scientists than their male counterparts. This result is consistent with previous findings [8, 56]. For example, Abramo et al. [56] concluded that a star scientist is “a typically male professor.” In another study which employed the pooled data from the United Kingdom it is shown that both employment and retention of women in science remains relatively low [35]. Another study surveyed scientists at elite universities and presented solid evidence in regard to the fact that the gender inequality of male and female scientists persists [57].

### Limitations and future research

Using a non-random sampling method to select participants was one of the methodological limitations of this study. Additionally, the cross-sectional nature of the research design precluded a deeper understanding of the relationship between study variables. Therefore, it may be useful to conduct a longitudinal study using mixed method approaches or experimental designs for a more comprehensive investigation of the research problem.

Although the study shows the important effects of cultural capital on being an elite scientist, these effects are not causal. Future studies can use real experimental designs to explore whether the effect of cultural capital on scientists’ performance is causal. Nevertheless, despite all these limitations, the current findings contribute to the studies on higher education by describing new relationships between structures.
